# Anatomical Variations of the Neurovascular Structures of the Hand and the Clinical Significance

**DOI:** 10.7759/cureus.99862

**Published:** 2025-12-22

**Authors:** Slobodan Kapor, Predrag Bjelogrlic, Valentina Simic, Drazan Eric, Suncica Kapor, Fraser Chisholm, Enis Cezayirli, Darko Laketic

**Affiliations:** 1 Department of Neuroanatomy, "Niko Miljanic" Institute of Anatomy, University of Belgrade School of Medicine, Belgrade, SRB; 2 Department of Clinical Skills, University of St Andrews, St Andrews, GBR; 3 Department of Anatomy, "Niko Miljanic" Institute of Anatomy, University of Belgrade School of Medicine, Belgrade, SRB; 4 Department of Plastic Surgery, Al Hammadi Hospitals, Doha, QAT; 5 Department of Internal Medicine, Clinical Hospital Center Dr Dragiša Mišović, Belgrade, SRB; 6 Department of Anatomy, University of St Andrews, St Andrews, GBR

**Keywords:** berrettini anastomosis, digital arteries, hand anatomy, kaplan’s line, nerve communication, superficial palmar arch

## Abstract

Background

Anatomical variations in the neurovascular structures of the hand have critical implications for surgical procedures, diagnostic accuracy, and nerve conduction studies. This study examined the morphology of the superficial palmar arch (SPA), the course of common digital palmar (CDP) arteries relative to Kaplan’s line, and the prevalence and types of Berrettini anastomosis.

Methods

Thirty-six embalmed cadaveric upper limbs (18 right, 18 left) from the Universities of Belgrade and St Andrews were dissected following standard anatomical protocols. Specimens with visible trauma or deformity were excluded. The SPA was classified as complete or incomplete. Origins of CDP artery distances from Kaplan’s line were measured and analyzed for sex differences using the t- test (p < 0.05). Median-ulnar nerve communications were identified and categorized as Type I (ulnar to median), Type II (median to ulnar), or Type III (bidirectional/multiple).

Results

Complete SPAs were observed in 55.6% (n=20) of specimens, predominantly in males, while 44.4% (n=16) showed incomplete patterns, more frequently in females. CDP arteries were significantly farther from Kaplan’s line in male specimens (p < 0.05). The Berrettini anastomosis was present in 33.3% (n=12) of hands, with Type III being most common (66.7%, n=8/12), and no Type I anastomoses were identified.

Conclusions

Our findings highlight clinically significant variations in hand neurovascular anatomy, with notable sex-based differences in SPA configuration and arterial topography. The frequent occurrence of complex Berrettini anastomoses underscores the importance of detailed anatomical awareness to avoid complications in hand surgery and to enhance diagnostic procedures.

## Introduction

The ulnar artery is a major vessel in the upper limb. It comes from the brachial artery near the cubital fossa and travels along the inner forearm before entering the hand. It passes above the flexor retinaculum near the pisiform bone, mainly supplying blood to the palm and fingers [1-3].

One of its main roles is forming the superficial palmar arch, an arterial network located above the flexor tendons and below the palmar aponeurosis. The superficial palmar arch can vary greatly in structure and completeness. It usually forms mostly from the ulnar artery, supplemented by a branch from the radial artery. However, there are cases where the arch is entirely made by the ulnar artery, or it may be incomplete. Sometimes, the median artery or other branches contribute as well [1,3,4]. These variations are categorized by completeness and the source of arterial supply, which is important for surgical planning to avoid unintended damage to blood vessels [5].

Nearby are the median and ulnar nerves, which provide movement and sensation to the hand. Both nerves create palmar digital branches and have connections between them known as neural anastomoses. The patterns and frequency of these connections differ widely, showing the complex wiring of the hand [6].

Several types of anastomoses between the median and ulnar nerves exist, including the Berrettini anastomosis, the Riché-Cannieu anastomosis, and the Martin-Gruber anastomosis. The Berrettini anastomosis is the most common palmar neural connection. It forms between the common palmar digital nerves of the ulnar and median nerves, usually found between the third and fourth digits [7,8]. The Riché-Cannieu anastomosis connects the recurrent branch of the median nerve with the deep branch of the ulnar nerve in the thenar muscles, impacting motor control [2,9]. The Martin-Gruber anastomosis connects the median and ulnar nerves in the proximal forearm, affecting motor fibers before they reach the hand [10].

Understanding these anastomoses is crucial because they affect sensory and motor functions, complicate nerve conduction studies, and can lead to unexpected clinical issues during neuropathies or surgeries. The Berrettini anastomosis, in particular, shows variation in its form and frequency and has been grouped by the direction, number, and complexity of its connections [11]. This study examines the detailed anatomical features and variations of the Berrettini anastomosis, along with the vascular structures of the ulnar artery and the superficial palmar arch, to enhance clinical and surgical knowledge.

## Materials and methods

This was an anatomical study using cadavers. The cadavers were obtained from the anatomical dissection hall of the Institute of Anatomy, School of Medicine, University of Belgrade, Serbia, and Department of Anatomy, School of Medicine, University of St Andrews. A total of 36 embalmed cadaveric upper limbs (18 right and 18 left) were used in this study.

Dissection process

First, a visual inspection of each limb was performed to exclude specimens with deformities and traces of trauma or surgery. After fixation in formalin solution, the extremities were carefully dissected, following standard procedures from dissection manuals. The limbs were placed in a supine position with the palm facing upward.

A longitudinal skin incision was made from the distal forearm to the middle of the palm. Skin and subcutaneous tissue were carefully reflected to expose the palmar aponeurosis. The palmar aponeurosis was incised and reflected to reveal the median and ulnar nerves and their common digital branches. The area between the third and fourth common digital nerves was closely examined. Any communicating branches between the median and ulnar nerves were noted. The origin, course, and termination of the anastomotic branch were traced and documented.

After identification of the median and ulnar nerves, we located the ulnar artery as it enters the palm, superficial to the flexor retinaculum (transverse carpal ligament), often just lateral to the pisiform bone. We traced the ulnar artery distally as it curves laterally across the palm, typically forming the superficial palmar arch, and identified contributions from the radial artery or occasionally the median artery joining to complete the arch. After identification of the SPA, we carefully cleared the connective tissue around the arch to fully expose its arc shape and preserved and noted the common digital palmar (CDP) arteries branching off the arch, supplying the fingers.

Kaplan’s line is an important anatomical landmark used in hand surgery and clinical anatomy to help localize structures on the palmar surface of the hand. It is an imaginary transverse line drawn across the palm, extending from the apex of the first interdigital space (the cleft between the thumb and index finger) toward the hook of the hamate bone on the ulnar side. It typically lies proximal to the distal palmar crease. Kaplan’s line serves as a guide to the safe zone during surgical approaches to the palm, particularly in procedures involving the carpal tunnel and the superficial palmar arch. In our study, after identification of the 1st-4th CDA, we measured its distance from Kaplan’s line [12,13] and statistically examined differences between male and female cadavers.

Statistical analysis

All statistical measurements were done in SPSS Statistics for Windows, version 11.0 (Released 2002; SPSS Inc., Chicago, Illinois, United States) using the t-test. p<0.05 was considered statistically significant.

## Results

**Figure 1:**
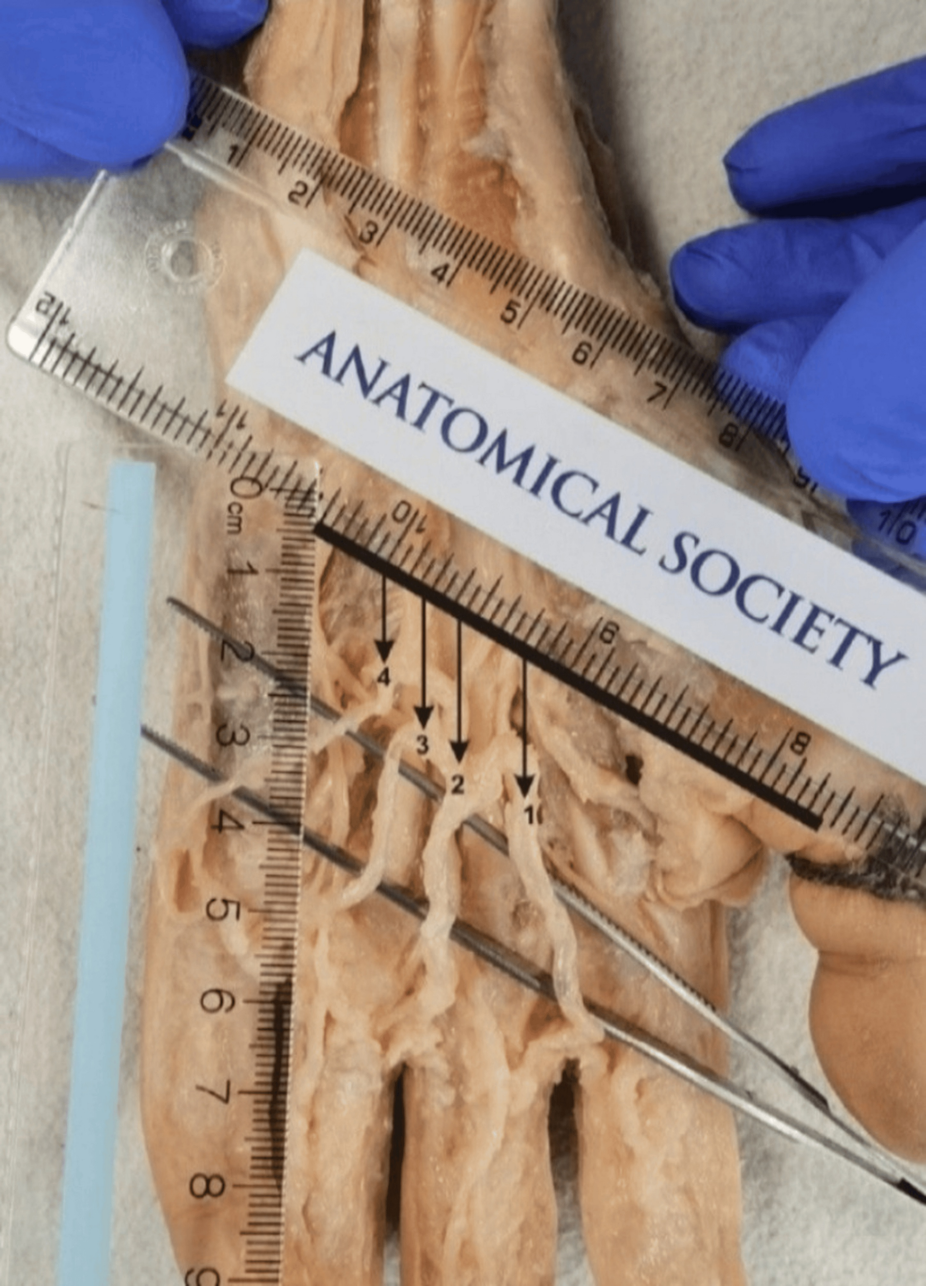
Methodology of examined parameters Kaplan‘s line is presented with a black, bolded line. Numbers 1-4 are CDP arteries, while the arrows show the distance between Kaplan’s line and the origin of the CDP artery from the superficial palmar arch. CDP: common digital palmar Dissection done by: Dr. Slobodan Kapor

**Figure 2:**
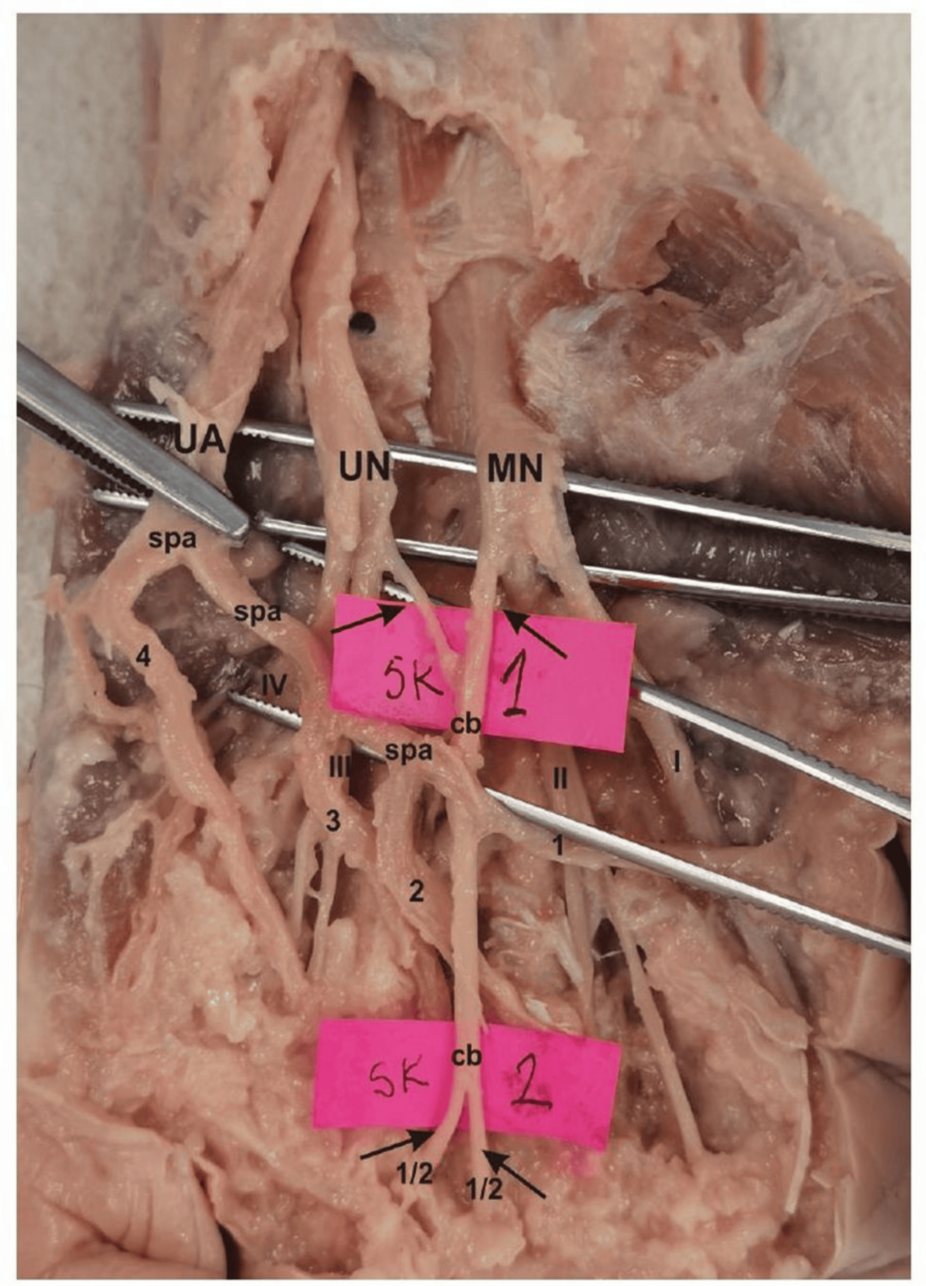
Dissection view of superficial palmar arch (complete type), as well as common palmar digital arteries and common digital palmar nerves with Berrettini anastomosis (type III) UA: ulnar artery; MN: median nerve; UN: ulnar nerve; spa: superficial palmar arch; cb: common bidirectional branch; 1-4: common digital palmar arteries ; I-IV: common digital palmar nerves Dissection done by: Dr. Slobodan Kapor.

**Figure 3:**
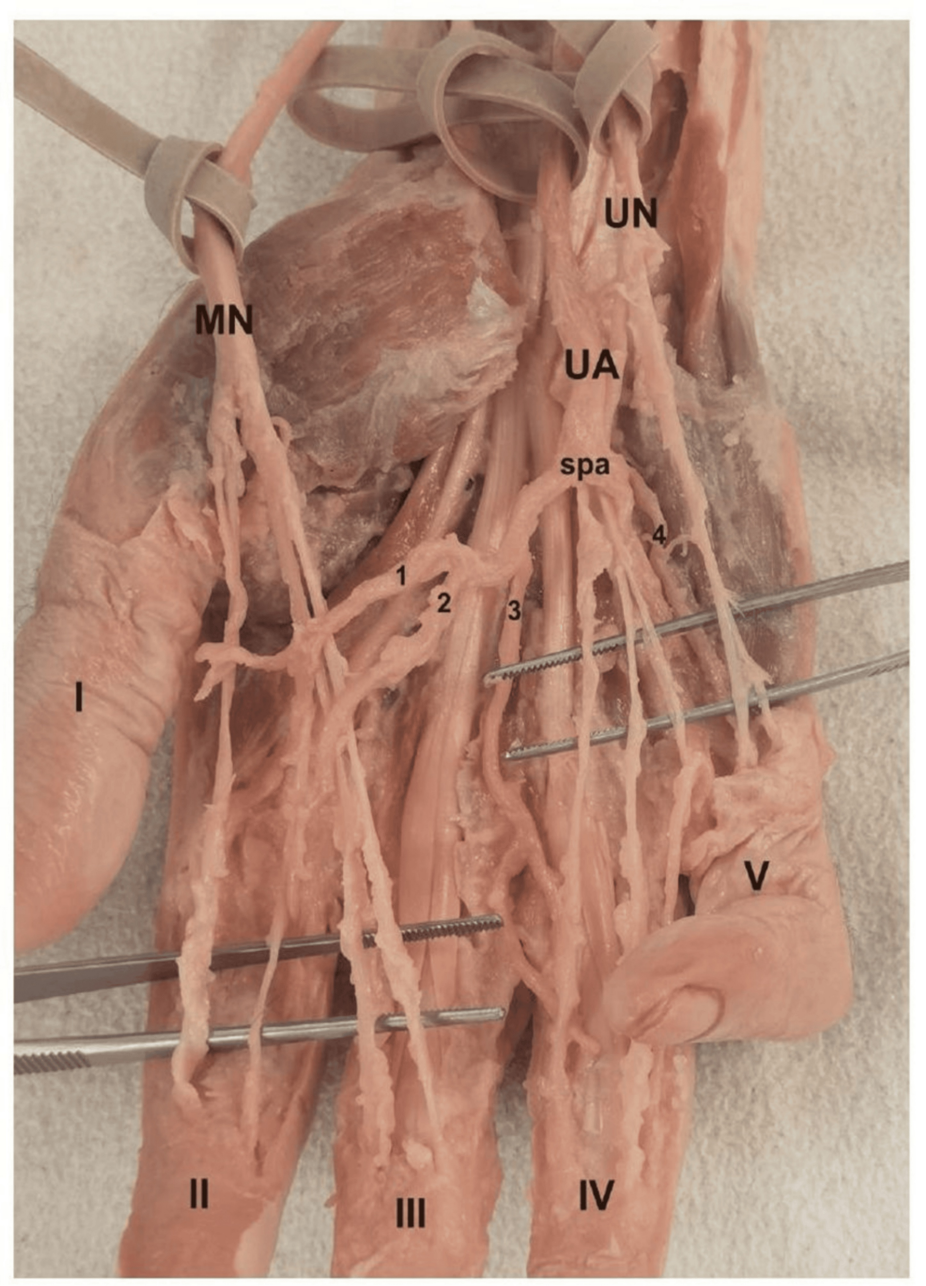
Dissection view of superficial palmar arch (complete type), as well as CDP arteries and CDP nerves with no Berrettini anastomosis Median nerve innervates I – III fingers, while ulnar nerve is responsible for innervation of last two fingers (IV-V). UA: ulnar artery; MN: median nerve; UN: ulnar nerve; spa: superficial palmar arch; CDP: common digital palmar Dissection done by: Dr. Slobodan Kapor.

We analyzed 36 cadaveric hands (20 female, 16 male) to assess variations in the SPA and the distribution of CDP arteries (Figures 1-3). Among the hands, 16 (44.4%) demonstrated incomplete SPAs, and 20 (55.6%) showed complete SPAs. A sex-specific distribution was noted: 12 of the 16 incomplete types were from female specimens, while four were from male specimens. Conversely, 12 of the 20 complete arches were from male specimens, with the remaining eight from female specimens. Thus, incomplete types were more prevalent among female specimens, while complete types were more common in males.

Distance from Kaplan’s line to CDP arteries

Measurements (in cm) from Kaplan’s line to the first through the fourth CDP arteries are presented in Table 1 and were significantly greater in male specimens than in female specimens (p < 0.05 for all comparisons).

**Table 1:** Measurements of the distance between the Kaplan‘s line and 1-4 CDP arteries *statistically significant value (p< 0.05) CDP: common digital palmar

Artery	Female (n=20), mean ± SD	Male (n=16), mean ± SD	p-value	t-test statistic
4th CDP	1.08 ± 0.17 cm	1.60 ± 0.15 cm	p< 0.05*	t= -7.56
3rd CDP	1.35 ± 0.17 cm	1.76 ± 0.15 cm	p< 0.05*	t= -6.06
2nd CDP	1.58 ± 0.13 cm	2.96 ± 0.15 cm	p< 0.05*	t= -6.56
1st CDP	1.81 ± 0.11 cm	2.21 ± 0.15 cm	p< 0.05*	t= -9.38

Berrettini anastomosis

The Berrettini anastomosis, a communicating neural branch between the median and ulnar nerves, was identified in 12 out of 36 hands (33.3%). We defined three types of communication between median and ulnar nerves: type I (ulnar to median); type II (median to ulnar); type III (multiple or bidirectional connections). These results are presented in Table 2.

**Table 2:** Tabular presentation of Berrettini anastomosis (N=36 hands)

Parameter	Total number	Percentage of 36 Hands	Percentage of 12 Positive Cases
Hands with any anastomosis (Type II/III)	12	33.3%	100%
• Type I (U → M)	0	0%	0%
• Type II (M → U)	4	11.1%	33.3%
• Type III (bidirectional/multiple)	8	22.2%	66.7%

## Discussion

This study investigated anatomical variations of the SPA, distances from Kaplan’s line to the CDP arteries, and the occurrence of the Berrettini anastomosis in 36 cadaveric hands. Our findings contribute valuable data on hand anatomy with potential clinical implications.

The prevalence of complete (55.6%) and incomplete (44.4%) SPAs in our sample aligns with previous literature, which reports SPA completeness rates ranging from 40% to 90% depending on the population studied and methodology used [13-19]. Some researchers observed complete arches in about 52.2% of hands, with 47.8% being incomplete [12], while Rodriguez-Niedenfuhr and colleagues reported complete SPAs in 50-60% of adult specimens [20].

The sex-related difference observed, with incomplete SPAs more common in females and complete SPAs more prevalent in males, has been reported less frequently [1,2,5,19,20], but was also noted by Tubbs et al. [7,8] as well as Zarzecki et al. [21], suggesting sexual dimorphism in hand vascular anatomy. This variation is important for surgical planning, as incomplete arches may increase the risk of ischemic complications during hand surgery, particularly in females.

Our data revealed significantly greater distances from Kaplan’s line to the CDP arteries in male specimens compared to female specimens (p < 0.05 for all arteries). This finding confirms previously described sexual dimorphism in hand anatomy, including vascular and neural structures [14]. Kaplan’s line remains a crucial landmark for surgeons to avoid vascular injury during procedures such as carpal tunnel release and digital artery dissection [12]. Although previous studies have measured distances of arterial branches in the palm, data specific to Kaplan’s line distances and sex differences are limited, underscoring the novelty and clinical relevance of our results [12,16-18].

Berrettini anastomosis was present in 33.3% of hands, consistent with prior studies reporting a prevalence between 20% and 70% [1,11,22]. The predominance of Type III anastomoses (66.7%) in our sample concurs with Zolin et al.'s classification, which highlights the frequent presence of multiple or bidirectional communications [10]. The absence of Type I (ulnar- to- median) connections in our sample may be due to population variation or sample size limitations. These neural connections have important clinical implications since they may affect sensory innervation patterns and complicate the diagnosis and treatment of median and ulnar nerve pathologies [15,16].

Knowledge of these variations is essential for surgeons performing nerve decompression or repair in the hand to avoid inadvertent injury. The study was limited by a modest sample size of 36 cadaveric hands and lacked ethnic diversity, which may limit the generalizability of findings. Future investigations combining anatomical, imaging, and electrophysiological techniques in larger, diverse populations would provide deeper insight into the functional significance of these anatomical variations.

## Conclusions

Our findings highlight clinically significant variations in hand neurovascular anatomy, with notable sex-based differences in SPA configuration and arterial topography. The frequent occurrence of complex Berrettini anastomoses underscores the importance of detailed anatomical awareness to avoid complications in hand surgeries and enhance diagnostic procedures.

## References

[1] Bilge O, Pinar Y, Ozer MA, Gövsa F (2006). A morphometric study on the superficial palmar arch of the hand. Surg Radiol Anat.

[2] Unver Dogan N, Uysal II, Karabulut AK, Seker M, Ziylan T (2010). Communications between the palmar digital branches of the median and ulnar nerves: a study in human fetuses and a review of the literature. Clin Anat.

[3] Standring S, Gray H, Anand N, Tunstall R (2021). Gray’s Anatomy: The Anatomical Basis of Clinical Practice. https://archive.org/details/graysanatomyanat0000unse_c5d4/page/n1583/mode/2up.

[4] Moore KL, Dalley AF, Agur AM (2023). Moore's Clinically Oriented Anatomy. https://search.worldcat.org/title/1260173449.

[5] Borthakur D, Kumar R, Singh S (2022). Variations in superficial palmar arch: case series with clinico-anatomical perspective. Medeni Med J.

[6] Coleman SS, Anson BJ (1961). Arterial patterns in the hand based upon a study of 650 specimens. Surg Gynecol Obstet.

[7] Tubbs RS, Shoja MM, Loukas M (2008). Anatomical landmarks to the superficial and deep palmar arches. Plast Reconstr Surg.

[8] Tubbs RS, Shoja MM, Loukas M (2016). Bergman's Comprehensive Encyclopedia of Human Anatomic Variation. https://onlinelibrary.wiley.com/doi/book/10.1002/9781118430309.

[9] Wali A, Ahmed R, Khan S (2017). Electrophysiological evidence of the Riche-Cannieu anastomosis in the hand and its diagnostic implications; 2 case reports. Clin Neurophysiol Pract.

[10] Zolin SD, Barros MD, Abdouni YA, Nascimento Vd, da Costa AC, Chakkour I (2014). Anatomical study of sensory anastomoses in the hand. Acta Ortop Bras.

[11] Formatted: Sharma, R. R., & Knipe, H. (2025 (2025). Radiopedia: Berrettini anastomosis. https://radiopaedia.org/articles/berrettini-anastomosis.

[12] Sunderland S (1968). Nerves and Nerve Injuries. 2nd ed. Elsevier.

[13] Panchal AP, Trzeciak MA (2010). The clinical application of Kaplan's cardinal line as a surface marker for the superficial palmar arch. Hand (N Y).

[14] Vella JC, Hartigan BJ, Stern PJ (2006). Kaplan's cardinal line. J Hand Surg Am.

[15] MacKinnon SE, Novak CB (1999). Nerve transfers: new options for reconstruction following nerve injury. Hand Clin.

[16] Kaur N, Singla RK, Kullar JS (2016). Cadaveric study of Berretini communications in North Indian population. J Clin Diagn Res.

[17] Hudson AR, Kline DG, Kim DH (2013). Atlas of Peripheral Nerve Surgery. Plast Reconstr Surg.

[18] Solmaz E, Fazliogullari Z, Albay S, Unver Dogan N, Karabulut AK (2023). Anatomical variations of the superficial palmar arch in human fetuses. Anat Sci Int.

[19] Ottone NE, Prum N, Dominguez M (2010). Analysis and clinical importance of superficial arterial palmar irrigation and its variants over 86 cases. Int J Morphol.

[20] Rodríguez-Niedenführ M, Burton GJ, Deu J, Sañudo JR (2001). Development of the arterial pattern in the upper limb of staged human embryos: normal development and anatomic variations. J Anat.

[21] Zarzecki MP, Popieluszko P, Zayachkowski A, Pękala PA, Henry BM, Tomaszewski KA (2018). The surgical anatomy of the superficial and deep palmar arches: a Meta-analysis. J Plast Reconstr Aesthet Surg.

[22] Smith JL, Siddiqui SA, Ebraheim NA (2019). Comprehensive summary of anastomoses between the median and ulnar nerves in the forearm and hand. J Hand Microsurg.

